# UK women’s experiences of breastfeeding and additional breastfeeding support: a qualitative study of Baby Café services

**DOI:** 10.1186/s12884-015-0581-5

**Published:** 2015-07-07

**Authors:** Rebekah Fox, Sarah McMullen, Mary Newburn

**Affiliations:** NCT, Alexandra House, Oldham Terrace, London, W3 6NH UK

**Keywords:** Breastfeeding, Breastfeeding support, Baby Café, Women’s experiences, Unrealistic expectations, Expert support, Social support, Breastfeeding role models

## Abstract

**Background:**

Whilst 81 % of UK women initiate breastfeeding, there is a steep decline in breastfeeding rates during the early postnatal period, with just 55 % of women breastfeeding at six weeks. 80 % of these women stopped breastfeeding sooner than they intended, with women citing feeding difficulties and lack of adequate support. As part of efforts to increase breastfeeding continuation rates, many public and voluntary organisations offer additional breastfeeding support services, which provide practical support in the early postnatal period and beyond. This paper focuses on the qualitative experiences of UK users of Baby Café services to examine their experiences of breastfeeding and breastfeeding support.

**Methods:**

The study was based upon in-depth interviews and focus groups with users of eight Baby Café breastfeeding support groups across the UK. Thirty-six interviews and five focus groups were conducted with a total of fifty-one mothers using the service. Interviews and group discussions were analysed using N Vivo software to draw out key themes and discussions.

**Results:**

Whilst each mother’s infant feeding journey is unique, reflecting her own personal circumstances and experiences, several themes emerged strongly from the data. Many women felt that they had been given unrealistic expectations of breastfeeding by professionals keen to promote the benefits. This left them feeling unprepared when they encountered pain, problems and relentlessness of early infant feeding, leading to feelings of guilt and inadequacy over their feeding decisions. Mothers valued the combination of expert professional and peer support provided by Baby Café services and emphasised the importance of social support from other mothers in enabling them to continue feeding for as long as they wished.

**Conclusions:**

The research emphasises the need for realistic rather than idealistic antenatal preparation and the importance of timely and parent-centred breastfeeding support, particularly in the immediate postnatal weeks. The findings suggest that effective social support, combined with reassurance and guidance from skilled practitioners, can help women to overcome difficulties and find confidence in their own abilities to achieve their feeding goals. However, further work is needed to make sure such services are readily accessible to women from all sectors of the community.

## Background

Exclusive breastfeeding until six months of age is recommended by the World Health Organisation [1] as providing short and long term health benefits for both mothers and children. Whilst UK rates of breastfeeding initiation have risen from 62 % in 1990 to 81 % in 2010 [2], there remains a steep decline in breastfeeding rates during the early weeks, with 55 % of women still breastfeeding at six weeks and just 34 % at six months. The UK lags behind other western countries such as in Scandinavia, where 80 % of Norwegian mothers [3] and 68 % of Swedish mothers [4] are still breastfeeding at six months. Rates of exclusive breastfeeding are even lower, with just 23 % of UK women exclusively breastfeeding at six weeks and only 1 % by six months [2]. Much of this drop-off is unplanned, with 80 % of women who stopped feeding during the first six weeks saying they would have liked to continue for longer [2].

Problems such as inability to latch, sore or painful nipples and insufficient milk supply are frequently cited reasons for early breastfeeding cessation [2]. Women are often unprepared for the physical challenges of early breastfeeding [5] and feel that they are left to ‘learn the hard way’ due to lack of time, expertise or practical assistance from health professionals [6]. However, the broader causes are more complex, with breastfeeding prevalence linked to demographic and socio-cultural factors, such as mother’s age, ethnicity, education, profession, level of deprivation and personal or familial breastfeeding experience [2, 7]. A long history of UK formula feeding and negative attitudes towards breastfeeding in public means that many women may not have had first-hand exposure to breastfeeding and may lack practical experience and support from relatives or friends [8]. Embodied experience of seeing friends or family successfully breastfeeding, or having breastfed a previous child, are important factors in motivating women to initiate and continue breastfeeding [8, 9]. Conversely women from communities where formula feeding is seen as the norm are more likely to view breastfeeding as difficult and potentially embarrassing [10, 11], and see the breast as a primarily sexual object [9]. For younger mothers, breastfeeding is often not widely accepted within their social circles and formula is seen as offering an ‘easier’ solution that enables them to share the burden of feeding with other family members [11, 12].

Health promotion messages focusing upon the ‘breast is best’ message are often perceived as prescriptive and unhelpful in the lived reality of everyday relationships, where competing goals of immediate family well-being are often valued over the long term health benefits of exclusive breastfeeding [13]. Mothers frequently experience conflicting advice and pressures from health professionals, partners, family and friends to conform to social norms and expectations of ‘good motherhood’ [14] whether this be supplementation with formula where the baby is perceived as not gaining adequate weight, or to continue breastfeeding despite personal discomfort. Idealistic representations of breastfeeding during the antenatal period may mean that women do not prepare for the need for postnatal support [15]. Unrealistic promotion of breastfeeding as ‘natural’ and unproblematic [15] can lead to feelings of failure if women do not experience the breastfeeding relationship as harmonious or pleasurable [16, 17]. Failure to continue breastfeeding can lead to ‘shattered expectations’ [18] and feelings of guilt and failure, in a society where breastfeeding is highly promoted in the public health agenda [13, 14]. Mothers perceive moral judgement on their feeding methods *regardless* of how they feed their babies, for example by ‘risking’ the health of their babies by formula feeding [12, 19] or exceeding limits of what some consider to be ‘acceptable’ breastfeeding behaviour, such as breastfeeding in public or for extended periods of time [20].

Whilst the health benefits of breastfeeding are now widely acknowledged, the lived reality of women’s everyday experiences means that idealistic feeding goals may be impractical [21]. Recent approaches to infant feeding support suggest the need to move away from ideas of one-off ‘choices’ to breastfeed to explore women’s experiences and decisions throughout their feeding journeys [15, 21]. A shift in health policy has been recommended away from breastfeeding promotion, and towards providing practical and ongoing ‘mother-centred’ feeding support in the early postnatal period and beyond [15]. The need to focus upon relationship building and practical breastfeeding support is now widely recognised in both research and policy [22, 23].

Various types of ‘additional’ breastfeeding support are currently available in the UK, including one-to-one support from midwives, health visitors, qualified breastfeeding counsellors or lay peer supporters [24, 25], breastfeeding support groups [26], and telephone helplines [27]. Evidence for the effectiveness of these interventions is mixed, with recent UK trials showing limited positive results [28]. However, evidence suggests that women often value these services and they provide pyscho-social benefits for the mothers, peer supporters and professionals involved [25]. A recent Cochrane review of 52 trials of support for healthy breastfeeding mothers [29] found an overall effect of extra breastfeeding support (including all forms, lay and professional) in increasing the duration of both exclusive and any breastfeeding. Face-to-face support was found to be more effective than telephone support, and proactive support was found to be more effective than interventions where the mothers were required to initiate contact themselves [29]. However a qualitative study of women’s experiences of group based or individual peer support found that women preferred a group-based approach which normalised breastfeeding, and provided flexibility and a greater sense of empowerment and self-control [30]. One-to-one peer coaching was perceived as more intrusive and a greater risk to self-confidence, and lacked the net social and interactional gains of a group situation [30]. A systematic review of evidence from randomised control trials concluded that peer support interventions did not have a significant effect on increasing breastfeeding rates in high income countries, particularly the UK [24].

Timing of support plays a key role, with evidence from a 2006 maternity survey [31] suggesting that additional support in the first ten days after birth has a significant effect on breastfeeding rates. The sharpest drop-off in breastfeeding occurs during the first two days, so support during this initial period is crucial [32]. Intensity of support is also important with evidence suggesting that interventions involving several contacts between mother and supporter are more effective than one-off episodes of care [24, 29]. Between four to eight contacts seems to provide an optimum effect [29], allowing the support to intervene at key points during the infant feeding journey [21]. Multi-channel interventions that are tailored to local contexts and populations, and involve both antenatal and postnatal contacts and continuity of care, are more likely to be effective [25, 33]. However the success of these interventions is dependent on local conditions, including health service resources, motivation of staff and integration with mainstream services [26]. Engagement of mothers with support services can also be problematic. Older, more highly educated mothers are more likely to seek help with breastfeeding difficulties [34], whilst pro-active approaches may be perceived as pressurising and impact on women’s sense of self-efficacy [15, 30].

This article focuses on the qualitative experiences of UK users of the Baby Café network of community based breastfeeding support services. These services offer a combination of expert one-to-one support from skilled professionals, combined with group-based social support from volunteers, peer supporters and other breastfeeding mothers. The paper examines women’s experiences of breastfeeding in contemporary Britain, support from health professionals, friends and family and the experience of accessing and using this particular model of breastfeeding support. The study identifies which elements of this support are seen to be effective and why, and considers how such services can be integrated with other forms of support to influence breastfeeding outcomes.

## Methods

### Settings and participants

The study was based upon in-depth interviews and focus groups with users of eight breastfeeding support groups, which were part of a UK network of ‘Baby Café’ services. The social model of support on which the service is based aims to combine mother-to-mother support in a welcoming, but protected, café style environment, with direct access to expert practical support for breastfeeding and referral pathways for additional clinical care where required. Services are delivered to a specific set of quality standards and group facilitators are either health professionals (e.g. midwives or health visitors) with specific experience or training in supporting breastfeeding women, or Association of Breastfeeding Mothers / Breastfeeding Network / La Leche League / NCT qualified breastfeeding counsellors, many of whom are also International Board Certified Lactation Consultants (IBCLC). Services are located in accessible community settings (e.g. children’s centres, health centres or community halls) and are available free of charge to all mothers needing support with infant feeding.

The study was designed and conducted in accordance with the Social Research Association’s ethical guidelines and research procedures. The study protocol was approved by the NCT ethical review committee prior to commencing the research, including review by two external academic advisors. An Evaluation Steering Group consisting of internal and external stakeholders, including support group facilitators, oversaw the conduct of the research, meeting four times throughout the duration of the project to advise on design, data collection and analysis.

The eight sites were selected to represent a variety of locations, settings, types of facilitator (health professional/breastfeeding counsellor) and length of time they had been running. Two sites were located in inner London, two in outer London, one in rural South East England, and one in a city and two in towns in Northern England. Support group facilitators were contacted in advance by the lead researcher (RF) to ask if they wished to take part in the project and other local staff informed of the research. In total nine sites were approached, with one declining to take part as the facilitator felt that their cramped location in a busy health centre would make it unsuitable for conducting potentially sensitive interviews.

Each site was visited separately on two occasions by the lead researcher (RF) to conduct individual interviews and focus groups. Participation was voluntary and respondents were given information sheets regarding the research and required to sign consent forms, understanding that all information would remain confidential and anonymous and they were free to withdraw from the research at any time. Thirty-six interviews and five focus groups (with between three and seven participants) were conducted with a total of fifty-one mothers using the service. Interviews and focus groups were chosen as suitable methods to elicit women’s feelings, behaviours and experiences. Individual interviews allowed for more in-depth exploration of sensitive issues, whilst focus groups were able to generate discussion of key issues and emerging themes.

The study was based upon convenience sampling and thus any mother present at the groups on the day of the visits was eligible to take part. The researcher used her judgement in not approaching women who were very obviously upset or struggling with acute breastfeeding difficulties, however several of these women did approach the researcher later in the session to speak about their experiences. In total sixty-three mothers were approached with fifty-one agreeing to participate. Mothers were given a choice of taking part in individual or group discussions (with seven taking part in both). The sample size was determined by availability of participants, with some Baby Cafés attracting a larger number of mothers than others. However at the end of the data collection period it was felt theoretical saturation had been reached and no further visits were required.

### Data collection

Interviews were conducted by RF during the support group sessions (n = 41) or by telephone at a later date (n = 6). Five focus group discussions were facilitated by RF, either in a separate room during the Baby Café session (n = 3) or in the main room after the session had ended (n = 2). Focus groups were not possible at three locations due to lack of time or space. Interviews were conducted in a busy support group environment and therefore tended to be relatively brief, lasting between 6 and 52 min (mean = 27 min). Interviews were sometimes disrupted or terminated prematurely by staff arriving to provide episodes of support to the women, or by the baby’s requirements. Focus groups lasted between 35 and 78 min (mean = 52 min) and were less prone to disruption.

Interviews were semi-structured, allowing mothers to lead the discussion and spontaneously raise topics of importance to them. The interview schedule covered a broad range of topics relating to mothers’ experiences of breastfeeding and breastfeeding support, including their expectations versus realities, positives and negatives of breastfeeding, problems they had encountered, support they had received from health professionals, friends and family, experiences of seeking ‘additional’ breastfeeding support and using Baby Café.

### Data analysis

All interviews and focus groups were digitally recorded and transcribed verbatim, then analysed using NVivo software [35] to draw out key themes and discussions. Transcripts were coded initially by RF using an inductive approach to draw out key themes emerging from the primary data. Transcripts were then re-read in more detail by members of the research team (RF, SM & MN) to refine these themes and coded further to produce higher level concepts emerging from the research. These codes were then checked by a second independent researcher VB (employed by NCT but not involved in the research project) and the interpretation of data discussed during face to face meetings and agreed between members of the research team (RF, SM & MN). Codes were cross-referenced to draw out common or contrasting examples and illustrative quotes to support the wider theories [36]. This type of analysis produces a rich qualitative description, detailing individual experiences and adding depth to inconclusive quantitative evidence on the effectiveness of breastfeeding support interventions.

## Results

Whilst each mother’s infant feeding journey is unique, reflecting her own personal circumstances and experiences, several themes emerged strongly from the data. Particularly for first time mothers, antenatal preparation was often described as setting unrealistic expectations of breastfeeding, leading to feelings of failure where this did not work out as expected. Both primiparous and multiparous women shared feelings of pressure, guilt and blame regarding their feeding experiences, and reported conflicting advice and varying levels of support from health professionals, friends and family. All women interviewed for the study were currently receiving additional breastfeeding support via Baby Café services and valued both the expert and social support provided, including the use of other mothers and peer supporters as ‘breastfeeding role models’.

In this section we firstly present the site and participant characteristics, before discussing these themes in more detail within the context of wider literature on the subject. The results are presented in two main sections: experiences of breastfeeding and experiences of additional breastfeeding support.

### Site characteristics

The sites selected for the research aimed to cover a variety of locations, types of facilitator and length of time they had been established. All groups ran weekly (excluding public holidays) and were staffed by qualified facilitators, trained peer supporters and volunteers. Further characteristics of each Baby Café are shown in Table 1 below.

**Table 1:** Characteristics of each Baby Café site

	Site 1	Site 2	Site 3	Site 4	Site 5	Site 6	Site 7	Site 8
Location	Nothern England	Northern England	Northern England	Inner London	Inner London	Outer London	Outer London	SE England
Venue	Children's centre	Private sector	Children's centre	Health centre	Community centre	Children's centre	Children's centre	Community centre
Facilitator	Midwife	Midwife IBCLC	Breastfeeding counsellor IBCLC	Health visitor	Breastfeeding counsellor IBCLC	Breastfeeding counsellor IBCLC	Health visitor IBCLC	Breastfeeding counsellor
Length of time Baby Café open	9 years	11 years	10 years	3 years	3 years	2 years	8 years	3 years
Length of weekly session	2 h	3 h	2 h	2 h	2 h	1.5 h	2 h	2 h
Number of women using the service during 2014	148	442	119	96	276	87	174	142

### Participant characteristics

The study included both 33 primiparous and 18 multiparous women aged between 23 and 44 (mean age = 35). Mothers tended to be highly educated and in employment and all but one were living with a partner. Ten participants were born outside of the UK. Unsurprisingly the support groups located in inner London attracted the widest diversity of ethnicities and nationalities, reflecting the characteristics of the local population. Participant characteristics are shown in Table 2 below.

**Table 2:** Participant characteristics

Mother’s age	19 & under	20-24	25-29	30-34	35-39	40+	
	0	2	10	8	27	4	
**Baby’s age**	0-7 days	8-14 days	2-4 weeks	4-8 weeks	9-12 weeks	3-6 months	6 months +
	1	1	2	5	10	15	18
**Ethnic identity**	Asian / Asian British	Black / Black British	White British	White Other	Other		
	5	2	37	6	1		
**Education**	No formal education	GCSE / equivalent	A Level / equivalent	Undergraduate degree	Postgraduate degree		
	0	1	11	20	19		
**Employment**	Full time	Part time	Self-employed	Student	Unwaged not seeking work	Registered unemployed	
	17	22	2	3	6	1	

### Experiences of breastfeeding

In this section findings in relation to experiences of breastfeeding are presented under four sub themes: antenatal education, realistic experiences, postnatal care and support from friends and family

### Antenatal education: Unrealistic expectations

In line with findings of previous studies, many of the women interviewed felt that they had been given unrealistic expectations of breastfeeding by professionals keen to promote its health benefits [6, 15, 18] and expressed anger about the lack of preparation they had been given for potential difficulties.

In the beginning I expected it to be easy, because when you go to the antenatal classes and you watch these videos and you think ‘oh yeah it comes naturally’, it looks so easy, but then when comes to the real baby, it’s not like that, you have to work at it (Mother, age 29, first baby)

It’s a complete shock, you know, and your emotions are all over the place. No-one tells you. They don’t tell you that breastfeeding can be so difficult and you don’t realise… Maybe no-one can tell you, maybe it’s just impossible to ever really prepare you for what it’s going to be like (Mother, age 38, first baby)

Dominant discourses that portray breastfeeding as ‘easy’ and ‘instinctive’ left mothers with feelings of guilt and inadequacy at their inability to master a supposedly ‘natural’ skill.

I just remember being on the postnatal ward and feeling like everyone around me had their babies firmly attached to their boob except me, I just had her screaming and I was thinking ‘Oh God, it’s all gone wrong, what am doing? Is it just me?’ I mean I’m an intelligent person. Millions of women feed their babies this way. Why am I finding it so difficult? (Mother, age 31, first baby)

Lack of control over the situation contrasted with women’s views of themselves and undermined their sense of self-efficacy.

I don’t know if it’s the era that we live in now that women, maybe because we’re having babies older and we’re having our careers before our children, we have different expectations of how things are supposed to work and we’re used to a life where we’re totally in control and now all of a sudden we’re not (Mother, age 36, first baby).

Such experiences underline the need for realistic antenatal education [15, 21] to adequately prepare mothers for the realities of breastfeeding and strategies for coping with the demands of a new baby.

If I knew what was to come or what I could expect to happen then I wouldn’t have freaked out so much. Because even with all the information that I had I was very, very unprepared, I wasn’t prepared for the pain, or the bleeding nipples, or the cluster feeding. I didn’t know that babies could feed for six hours! I just didn’t know it was possible (Mother, age 28, first baby)

### Realistic experiences: Pressure, guilt and blame

Women felt that there was still a great deal of pressure upon mothers to breastfeed, but that this was not always backed up by adequate practical support. This led to guilt about making the ‘correct’ feeding decisions and how to do the ‘best’ for their baby [37].

I think there is a great deal of pressure, you know ‘breast is best’ and all that and I couldn’t help but feel that I was sort of, I wasn’t doing my job properly, if I didn’t at least give it my absolute best shot (Mother, age 34, first baby).

I think it took four or five weeks for her to regain her birth weight….and you kind of feel really guilty, don’t you? Like maybe I’m making the wrong choice here – maybe I’m being selfish by not giving her a bottle (Mother, age 30, second baby).

Mothers felt they had to justify their decisions in order to maintain a sense of their own moral position as a ‘good mother’ [14, 38].

You get the feeling that mixed feeding is kind of looked down upon, but I just felt I wasn’t producing enough milk, he was a big baby and he was always hungry, I felt it was the right thing to do (Mother, age 37, first baby).

Mothers may also frame their feeding decisions within a context of ‘biographical repair’ [38] perhaps making up for previous ‘failed’ feeding experiences.

My first baby I was feeding for one month. I didn’t have the support at the time. I was twenty and far away from my family. I couldn’t understand the pain. Now I am older and more mature. I know that the baby is more important than the pain, somehow it’s easier (Mother, age 25, second baby)

I know it sounds silly but I’ve got a great sense of pride and achievement because I didn’t manage so well with [Baby 1] and I’ve managed a lot better with [Baby 2]. I feel like I’m doing better for my baby because I know it’s the best thing for him. I know that I’m providing that. When I stopped early last time I felt like I’d let him down (Mother, age 24, second baby)

### Postnatal care: Conflicting advice and undermining of confidence

Many women expressed dissatisfaction with routine postnatal care, reporting that advice was often inadequate, contradictory and undermined their confidence in their feeding abilities [6, 39, 40]. In particular the notion that ‘breastfeeding shouldn’t hurt’ was found to be unhelpful where women were experiencing problems with sore and cracked nipples.

In the hospital they kept repeating that it shouldn’t be painful, if you are doing it right it shouldn’t hurt. And that wasn’t particularly helpful, because it was painful for me (Mother, age 32, first baby)

‘Disconnected encounters’ [41] with health professionals could leave women disheartened and afraid to ask for further support.

They said “Oh if you’ve got any issues, just call us”, so I called somebody and the lady that came, she wasn’t a midwife, she was a nursery nurse or something, this older lady and she just kind of snapped at me “Well of course of hurts if you’ve never done it before!” (Mother, age 28, first baby)

Staff could give very contradictory messages, promoting breastfeeding on the one hand, but also quick to resort to alternative feeding methods.

I think they’re understaffed in hospitals, they just don’t have the time to sit with you, and it takes a lot longer to spend the time to get you breastfeeding, rather than handing you a bottle…(Mother, age 33, first baby)

The midwives, obviously, are very focused on the babies putting on weight and not having to go back into hospital, so they just push you to top them up with formula (Mother, age 24, second baby)

Mothers felt that they had to be very determined to achieve their feeding goals in the face of pain and limited support, often relying on their own embodied knowledge that something ‘wasn’t right’.

They weren’t doing home visits on Christmas day so I just felt totally abandoned. I took her back into hospital on Boxing Day and insisted that they helped me. I thought things weren’t right with her… she wasn’t feeding and kept getting really upset. I kept on calling them and asking them to come and help me, but you have to just be so determined to do it. It’s a real struggle (Mother, age 31, first baby).

Such experiences emphasise the need for adequate resources and training of healthcare staff to support breastfeeding women and the importance of mother-centred and personal interaction skills [15]. Women particularly valued professionals who were supportive, non-judgemental and enabled them to make their own decisions and build upon existing support from friends and family [13, 41].

The midwives in hospital were really good because I just felt like I wasn’t alone and they kind of supported my husband to support me, he helped me once we got home with positioning and, you know, he would say, oh you remember about this position, why don’t you try that sort of thing (Mother, age 33, first baby)

Women also valued the opportunity to build personal relationships, emphasising the need for continuity of care in the antenatal and postnatal periods [25, 33].

I think my midwife has been very supportive and very helpful, she was the midwife that happened to be doing the antenatal classes that we went to in the NHS so that was good because we’d already developed a relationship. She was also a very practical midwife, so it was actually the sort of person I needed to say it quite bluntly on occasions (Mother, age 31, first baby)

### Support from friends and family

It is well recognised that mother’s immediate social and cultural circles have a strong influence on her decisions to initiate and continue breastfeeding [8, 9, 21]. Participants in the study came from a variety of cultural and social backgrounds with different histories of infant feeding practices. Mothers whose immediate family and friends had not breastfed themselves, or had bad experiences of breastfeeding, were sometimes unsupportive of their decision, or attempted to undermine their efforts.

When I first tried to breastfeed my mother was quite shocked because she didn’t feed, she doesn’t agree with the idea of breastfeeding, you know like ‘if I was meant to breastfeed, to have something sucking on my nipples, then I would have been born a cow’ (Mother, age 27, first baby)

All me friends were formula fans, and they were all like ‘oh, just stop doing it’ I was like ‘oh no, I want to carry on’. But everything from friends was like ‘you can stop, you can stop … just get some get formula and you can stop’ (Mother, age 29, first baby)

Even women who may initially have felt social pressure to breastfeed found that once they reached a certain point in their feeding journey, there was an equal pressure to ‘move on’ to formula as part of a ‘normal’ progression, highlighting continued negative conceptions in UK society of women who breastfeed for extended periods of time [20].

I think, in my NCT group now, there’s only one other mum still breastfeeding and they all say, you know, if you give her formula, you’ll get a better night’s sleep, and that kind of thing, so it’s hard to…there’s pressure on people to breastfeed, but there’s also pressure on breastfeeding mums not to… (Mother, age 34, first baby)

I don’t have any family around here, apart from [partners] family. They’ve been really unhelpful, to the point where I’ve got really frustrated with them ‘Oh, she’s still having it, she’s six months now, you don’t have to breastfeed her, she just needs her food now’ ‘why are you still breast feeding her all the time?’ (Mother, age 26, first baby)

Other mothers found that partners, friends and family had been key in supporting them to keep going when they encountered breastfeeding issues.

Well, in the end it was my mum, when I was having problems, she remembered the problems that she’d had feeding me, because I was her first, and that the only thing that had got it started for her was nipple shields, so she went out and bought some nipple shields for me, because no one had suggested that I try them. No one at all. But that was what I needed to actually get it to work (Mother, age 31, first baby)

However even supportive partners and other family members could question mother’s determination to breastfeed where they perceived the mother as not coping well or were concerned about her welfare.

My in-laws were going ‘give her a bottle, it’s not worth it’ and [partner] was so worried about me. There was a day when we got the breast pump and I was sat in the bed in tears, and [partner] was knelt beside me trying to express milk from my left boob, and I’ve got her on the other boob and my nipples were red raw bleeding…every time I could see her coming, I pulled back a bit, and it killed, it was so painful…but we wanted to do it such a lot, we just kept going (Mother, age 26, first baby)

Feeding decisions are not individual one-off choices but situated within mothers personal social and cultural situations, with various ‘significant others’ [13] playing an important role in decisions about breastfeeding initiation and continuation [15]. Therefore it is important to continue to work to change wider cultural perceptions of breastfeeding and offer ‘family-centred’ support that works within women’s own social networks to support them at pivotal points within their feeding journeys [13].

### Experiences of ‘additional’ breastfeeding support

In this section we present the results relating to women’s experiences of additional breastfeeding support provided by Baby Café services. The results are presented under five sub-themes: seeking breastfeeding support, expert support, social support, breastfeeding role models and breastfeeding as a journey.

### Seeking breastfeeding support

All women involved in the study were attending a Baby Café breastfeeding support group at the time of the interviews and were therefore likely to be those who were more determined to breastfeed and possessed the ‘social capital’ [42] to seek support and advice. Some of the mothers had already sought support from other services such as telephone helplines.

What I did beforehand was I rang the Breastfeeding Helpline and they were really, really good as well, and that’s kind of what made me think you know what, there’s different responses to how you feed your baby, conventional formula versus breastfed, and I thought I need to find a place to go to where I’ve got likeminded people and that grew my confidence to be honest as a new mum and as a breastfeeding mum (Mother, age 30, first baby)

Whilst contact with other breastfeeding support services may sometimes have been the impetus to attend, women valued the face-to-face element of contact with a skilled breastfeeding professional [29].

You need that one to one; you need to see somebody face to face. Sometimes, doing it on the phone, what you’re telling somebody, it might not be exactly how it is…Somebody like [group facilitator] can say ‘Well, actually, I can see that…What you need to do is change this, or that…you just can’t get that on the phone… (Mother, age 35, first baby)

Where Baby Cafés were well embedded within the local health service, or combined with other forms of support such as peer supporters on hospital wards, women often found it more straightforward to seek support [25].

Well you got a leaflet about all the support groups from the hospital and when the health visitor came to have a check she mentioned the group and told me about how good it is, so when I was struggling I thought I’d try it out (Mother, age 35, first baby)

It was one of the Breast Buddies (peer supporters) at the hospital who suggested it, because I was saying to her ‘oh, I think I’ve had enough…I think I’m just going to give up and go onto bottles’ and she was like ‘no no no, go to the Baby Café and have a chat with them first’ (Mother, age 27, first baby)

Convenience was also a factor, with women often preferring services in a familiar location that they may be visiting for other purposes.

I’ve heard about this place. I knew things were going on here, because I used to come and see my midwife next door. And they have other groups going on so you can just pop in and say hello, it’s not just all about the breastfeeding (Mother, age 30, second baby)

Many of the women were initially anxious about attending a group situation and unsure of what to expect.

I was nervous the first time, yeah. Not knowing what it were like…I thought it would be like a café in the middle of town, that was my visualisation of it, you know, and all these people sat there. It’s just the unknown I suppose, you’re not really sure what to expect (Mother, 27, first baby)

You’re tired because you’ve only just given birth and your child can’t feed and you know, you’re suddenly, it’s the very first time you’re supposed to just get your boobs out in public*…*it can be quite nerve-wracking and yeah, I totally see why people are put off to be honest (Mother, 30, first baby)

Reticence about first attending may particularly affect younger or less confident mothers, who might feel that they do not ‘fit in’ with the other mothers.

I was feeling I had the baby too early. I just look around and there was old mothers at mature age. I was really feeling, not maybe embarrassed, but anxious about them. Like maybe I shouldn’t be here, I’m never going to find any mother in my age (Mother, 25, second baby)

However once they had made the initial effort mothers often felt that the group provided a supportive environment to increase their sense of breastfeeding self-efficacy.

I think it gives you a sort of safe haven to start if you’re a bit uncomfortable about doing it in public, to do it where other people understand what it’s like to feel self-conscious and to struggle and, you know, but it’s still a public place and so you get that bit of confidence (Mother, age 31, first baby)

### Expert support

Reasons for attendance ranged from appreciating general support and reassurance to needing help with breastfeeding ‘crises’, where women often felt on the brink of being unable to continue.

I can honestly say at ten days old, I hadn’t slept, I was full of milk, [baby] was screaming, I walked in and just sat there and cried for two hours and just said if somebody doesn’t sort this out I am going home and I am going to give her a bottle (Mother, age 29, third baby)

For some women the visit to the Baby Café was seen as a ‘turning point’ in their breastfeeding relationship.

I was struggling for him to latch on. Couldn’t get any help from anywhere, I was absolutely end of my tether, beside myself, and on the verge of giving up, so I got a friend to bring me up here and [facilitator] took one look at him and diagnosed a tongue tie, arranged for me to have it snipped and gave me some tips on positioning. Within minutes, I thought ‘You know, actually, I think I can do this’ (Mother, age 36, first baby)

A metasynthesis of women’s perceptions and experiences of breastfeeding support found that women valued an ‘authentic presence’ where they developed a trusting relationship or rapport with the supporter, who took time to ensure their needs were met, provided an empathetic and caring approach and affirmation of the mothers own abilities [41].

I’ve always got the help. Always. No matter how silly the question is, they’ve always got an answer. And its nice because they do remember your name, they do remember your baby, and it just feels, it feels nice (Mother, 35, first baby)

Expert knowledge and experience is also seen as key, with women valuing the presence of a skilled facilitator [30].

I think the advice you get here is so much better than that you get from GP’s or health visitors, who sometimes don’t seem to know that much about breastfeeding. They are experts in their field and they are mums themselves, which is always, experience speaks volumes (Mother, age 34, first baby)

### Social Support

The informal atmosphere of Baby Café and the provision of refreshments were seen as providing a more socially acceptable environment in which to discuss their concerns [30].

I was a bit kind of apprehensive when I first started coming along ‘cos I don’t know, I don’t trust Health Visitors so I was thinking it would be kind of a bit kind of clinical because it was held in a health centre. Yeah, but I was just really pleased the first day I came, I really enjoyed it and it just felt really welcoming (Mother, age 33, first baby)

I think that the minute you walk in and somebody says ‘hello’ with an open arm and a cup of tea and a biscuit and when you’re a new mum and you’re not sleeping well, you’ve got feeding issues, you’re tired, you just want somebody to say ‘would you like a cup of tea and tell me all about it’ (Mother, age 31, first baby)

Involvement of partners or other supporters also increased mother’s sense of self-confidence.

We thought fathers were not allowed to stay here, but then [facilitator] said ‘no, we welcome dads as well’ so…he stayed and was chatting to everyone, and I felt really comfortable (Mother, age 29, first baby)

Social support from other mothers was seen as an important component of the service, providing benefits over and above ‘professional’ assistance with breastfeeding [30].

To be honest, most of the support I get on a day to day basis is from other mums. That’s been the key support in getting through the first three months, just getting out and seeing people (Mother, age 32, first baby)

This was particularly the case for women who were isolated from friends and family.

I find it really helpful because there’s nobody else to ask…I mean all my family’s back in Sri Lanka, so I don’t have my mum or a sister whatever to rely on, to ask for simple things, because when it’s your first child you don’t know what to do (Mother, age 35, first baby)

### Breastfeeding role models

The social model of the service meant that women could attend regularly if they wished and created a space for the sharing of infant feeding experience. Mothers found it helpful to be around other breastfeeding women, especially those with older babies who were further on in their feeding journey. This role modelling had a positive impact on those women who were still at the stage of getting breastfeeding established, and gaining confidence about feeding in public places;

I do think it’s quite important, that here, you can speak to other mums with older babies and see it does get better, because if you’re all sat here with newborns, all crying, all saying you can’t do it. You want to see that it will get better, to speak to a mum that says its better (Mother, age 30, first baby)

I got really nice help from the previous peer supporter that was here. She had an older baby and when you have a younger baby, it was nice to see an older baby breastfeed successfully…Oh! She does it with such comfort, makes it seem so effortless. It was a really nice thing to see… (Mother, age 34, first baby)

Such environments also played an important role in normalising the feeding of older babies, for mothers who wished to follow the guidance of complimentary breastfeeding for two years and beyond [20].

I kind of want to go past a year now and to come here and meet other people who like openly are like “Oh yeah, I’m still feeding my three year old” I’m a little bit like “Are you? Okay, great”. I’m shocked, I will admit it, I’m a little bit shocked, but I’m also like “Okay, other people are doing this, I can do it too” (Mother, age 30, first baby).

### Breastfeeding as a journey

Many of the women interviewed for the study were already in later stages of their breastfeeding journey and looking back upon the initial ‘investment and adjustment’ period [15] with the benefit of hindsight. This gave them a chance to reflect upon their feeding journeys, considering what had made a difference at pivotal moments.

I think in the grand scheme of things they weren’t really anything too major, it was all major in my own head because the pain was unbearable so like latching, unlatching, being in the shower was extremely painful, the water touching…and then of course, you know, he was feeding around the clock, you know, hour and a half for a feed, you know, your whole life is breastfeeding. But once you feel more confident, I think groups like this encourage you to just feed your baby in public, that kind of thing and once you know that you can go out and feed your baby then you can get your life back again (Mother, age 30, first baby)

Mothers found that they had often made peace with their feeding decisions and overcome some of the early challenges.

It’s got a lot easier now, but he’s still not having enough, so I give him the bottle as well. But I’m still breastfeeding, so it’s going well. He latches on well. He’s happy and I’m happy (Mother, age 32, first baby)

However breastfeeding constantly brings new challenges, for example in relation to weaning or returning to work, emphasising the importance of ongoing support [23]. Such support is necessary to enable women to meet (or even extend) their breastfeeding goals, and increase continuation rates in line with WHO standards [1].

I was always dead set on breastfeeding, but I’ve gone through spits and spurts of ‘Oh can I keep this up?’ because it is quite a demanding role, and coming here has made me think I actually can, because you can see all the mums here, you can speak to them. I’d always said ‘Right, when she goes into her own room at three months then I’ll stop’, but I haven’t stopped, I said ‘At six months I’ll stop’, and she’s almost that, I’m not planning to stop. My next goal’s a year (Mother, age 29, first baby)

## Discussion

The research shows that despite continued efforts to increase UK breastfeeding initiation and continuation rates, mothers still face substantial social, cultural, practical and physical barriers to successful breastfeeding [2, 13, 20]. Whilst policy and best practice such as the UNICEF Baby Friendly Initiative are steadily improving standards of care for breastfeeding mothers in UK hospitals [22], understaffing and lack of resources mean that women are not always able to access the support that they require from routine health care [6]. Prevalence of formula feeding amongst recent generations means that women may lack cultural support or practical breastfeeding expertise within their immediate social network [8, 9]. Antenatal contacts that set unrealistic expectations of the breastfeeding relationship can leave mothers unprepared for the lived experience of the early weeks and common breastfeeding problems [15, 18].

Whilst evidence on the effectiveness of ‘additional’ breastfeeding support interventions is limited [24, 28, 29], qualitative findings suggest that these can have a positive impact on women’s infant feeding journeys and contribute to the duration and quality of their feeding experiences [30, 31]. This study has focused upon one particular form of community-based support provided in the form of weekly Baby Café drop-ins facilitated by skilled health professionals or breastfeeding counsellors, with the help of trained peer supporters. Findings suggest that women value the social aspect of the Baby Café service and gain a great deal from interactions with other breastfeeding mothers, as well as from specialist expertise to address specific feeding difficulties [30]. Group environments normalise breastfeeding and can contribute to increased duration, providing ongoing support and assistance during the ‘pivotal points’ in a woman’s infant feeding journey [13] and enabling them to form continuing relationships.

However breastfeeding groups such as Baby Café tend to attract older, more advantaged mothers and those with a strong initial commitment to breastfeeding [30]. Motivation to breastfeed can be an important factor in accessing support services, with evidence from the 2010 Infant Feeding Survey [2] showing that whilst the majority of women received information about voluntary breastfeeding organisations, only a minority accessed them. The women interviewed for this study showed a strong commitment and determination to breastfeed, often continuing despite initial or ongoing difficulties and possessing both the motivation and ‘social capital’ to seek out additional support.

Conversely those women who lack initial commitment to breastfeeding or role models amongst their immediate social circles may be more likely to cease breastfeeding rather than proactively seek support. Group environments may appear daunting to younger or less confident women, particularly where they perceive themselves as not ‘fitting in’, or lacking in confidence to breastfeed in public or seek professional advice. Thus such interventions ideally need to combined with alternative forms of support, including proactive contact from peer supporters in the antenatal and immediate postnatal periods [25], and additional telephone and one-to-one support where required. Community support services need to be well integrated with local health and social care systems and promoted effectively to ensure that all women are able to access support when they need it.

### Strengths and limitations

The strengths of this research are that it provides an in-depth qualitative examination of women’s experiences of breastfeeding and using a particular form of community based breastfeeding support (Baby Café), adding depth to inconclusive evidence on the effectiveness of such interventions from UK randomised control trials.

The limitations of the study include a risk of bias due to the researchers being employed by NCT rather than an independent evaluator. This risk is minimised through the active involvement of external academic advisors on NCT’s Research Advisory Group, who have commented at all stages of the research and the fact that the research team are not involved in the delivery of NCT or Baby Café services. However it is recognised that their position within the organisation may influence their perceptions of breastfeeding support.

The research was conducted within a Baby Café setting, with group facilitators present (though not directly involved in the interviews / focus groups). Therefore mothers may have been less likely to report negative opinions or experiences of Baby Café services. The study sample is not intended to be representative of UK mothers as a whole, but of those accessing a particular support service. While the sample is diverse in terms of ethnicity, first language and place of birth, it is biased towards older mothers and those with higher levels of education and employment. Such groups are a) more likely to initiate breastfeeding, b) more likely to seek out support for breastfeeding difficulties and c) more likely to be willing to be involved in research interviews. Thus findings of the research cannot be generalised to the overall population and highlight the need to attract a more diverse range of mothers to such services or find alternative means of support for mothers from less advantaged populations.

## Conclusion

The research emphasises the need for realistic rather than idealistic antenatal preparation and the importance of timely and parent-centred breastfeeding support, particularly in the immediate postnatal weeks. The findings suggest that effective social support, combined with reassurance and guidance from skilled practitioners, can help women to overcome difficulties and find confidence in their own abilities to achieve their feeding goals. However, further work is needed to ensure that services such as Baby Café are readily accessible to women from all sectors of the community.
